# Serum immunoglobulin G and mucosal immunoglobulin A antibodies from prepandemic samples collected in Kilifi, Kenya, neutralize SARS-CoV-2 *in vitro*

**DOI:** 10.1016/j.ijid.2022.11.041

**Published:** 2023-02

**Authors:** James Nyagwange, Bernadette Kutima, Kennedy Mwai, Henry K. Karanja, John N. Gitonga, Daisy Mugo, Yiakon Sein, Daniel Wright, Donwilliams O. Omuoyo, Joyce U. Nyiro, James Tuju, D. James Nokes, Ambrose Agweyu, Philip Bejon, Lynette I. Ochola-Oyier, J． Anthony G. Scott, Teresa Lambe, Eunice Nduati, Charles Agoti, George M. Warimwe

**Affiliations:** 1 KEMRI-Wellcome Trust Research Programme,PO Box 230, Kilifi, Kenya; 2 School of Public Health, Faculty of Health Sciences, University of the Witwatersrand, 27 St Andrews Road, Parktown 2193, Johannesburg, South Africa; 3 Nuffield Department of Medicine, University of Oxford, Oxford OX3 7BN, United Kingdom; 4 The Zeeman Institute for Systems Biology and Infectious Disease Epidemiology Research (SBIDER), University of Warwick, Coventry, CV4 7AL, United Kingdom; 5 School of Life Sciences, University of Warwick, Coventry CV4 7AL, United Kingdom; 6 Department of Infectious Diseases Epidemiology, London School of Hygiene and Tropical Medicine, Keppel Street WC1E 7HT, London, United Kingdom

**Keywords:** SARS-CoV-2, Human coronaviruses, Pre-existing antibodies, Spike proteins

## Abstract

**Objectives:**

Many regions of Africa have experienced lower COVID-19 morbidity and mortality than Europe. Pre-existing humoral responses to endemic human coronaviruses (HCoV) may cross-protect against SARS-CoV-2. We investigated the neutralizing capacity of SARS-CoV-2 spike reactive and nonreactive immunoglobulin (Ig)G and IgA antibodies in prepandemic samples.

**Methods:**

To investigate the presence of pre-existing immunity, we performed enzyme-linked immunosorbent assay using spike antigens from reference SARS-CoV-2, HCoV HKU1, OC43, NL63, and 229E using prepandemic samples from Kilifi in coastal Kenya. In addition, we performed neutralization assays using pseudotyped reference SARS-CoV-2 to determine the functionality of the identified reactive antibodies.

**Results:**

We demonstrate the presence of HCoV serum IgG and mucosal IgA antibodies, which cross-react with the SARS-CoV-2 spike. We show pseudotyped reference SARS-CoV-2 neutralization by prepandemic serum, with a mean infective dose 50 of 1: 251, which is 10-fold less than that of the pooled convalescent sera from patients with COVID-19 but still within predicted protection levels. The prepandemic naso-oropharyngeal fluid neutralized pseudo-SARS-CoV-2 at a mean infective dose 50 of 1: 5.9 in the neutralization assay.

**Conclusion:**

Our data provide evidence for pre-existing functional humoral responses to SARS-CoV-2 in Kilifi, coastal Kenya and adds to data showing pre-existing immunity for COVID-19 from other regions.

## Highlights

• SARS-CoV-2 spike reactive serum immunoglobulin G antibodies are present in prepandemic samples.
• SARS-CoV-2 spike reactive mucosal immunoglobulin A antibodies are present in prepandemic samples.
• SARS-CoV-2 spike reactive prepandemic antibodies neutralize pseudotyped SARS-CoV-2.
• Prepandemic antibodies are also cross-reactive to human endemic coronaviruses.

## Introduction

SARS-CoV-2 emerged in 2019 and has caused morbidity, mortality, and disruptions in the global economy [1]. SARS-CoV-2 is a single-stranded RNA betacoronavirus in the *Coronaviridae* family that includes four human endemic coronaviruses (HCoVs): two betacoronaviruses, HCoV-OC43 and HCoV-HKU1, and two alphacoronaviruses, HCoV-NL63 and HCoV-229E, which are all associated with mild forms of respiratory infections; although, they can lead to severe disease in individuals with compromised immunity [2,3]. HCoVs are endemic in the human population and may be responsible for prepandemic SARS-CoV-2 cross-reactive T cell immunity and humoral immunity [4], [5], [6], [7], [8]. Pre-existing HCoV antibodies cross-reactive to SARS-CoV-2 are of great importance to COVID-19 progression and they have been reported in most settings as providing protection against COVID-19 [7,9,10] and in a few settings, as increasing COVID-19 pathogenesis possibly through the original antigenic sin phenomenon [11]. We recently reported about 10% spike reactive prepandemic serum at 1: 800 dilution in blood donors [12], and in the current study, we aimed to investigate the spike reactive prepandemic serum at lower dilutions in detail. We tested prepandemic antibodies in serum and naso-oropharyngeal (NP/OP) fluid collected in Kilifi, Kenya for HCoV binding and SARS-CoV-2 neutralization.

## Methods

### Study samples

The prepandemic serum samples (adults, n = 195 and children aged ≤15 years, n = 431) were from biobanked KEMRI-CGMRC annual cross-sectional surveys for malaria surveillance in coastal Kenya in 2018. The positive control was a pool of serum from 50 Kenyan adults with COVID-19 symptoms and SARS-CoV-2 reverse transcriptase-polymerase chain reaction (RT-PCR)-confirmed NP/OP samples.

The prepandemic NP/OP samples (n = 786) were obtained from biobanked human NP/OP samples collected from study participants between 2015 and 2017 in the Kilifi Health and Demographic Surveillance System. The pandemic NP/OP samples set (n = 1115) were SARS-CoV-2 RT-PCR test samples performed at the KEMRI-CGMRC between March and July 2020. Samples from both populations were collected using similar flocked nasopharyngeal swabs.

### Recombinant antigens production

We have recently described the production and purification of full-length SARS-CoV-2 spike protein in the mammalian expression system [12]. Recombinant spike antigens to HCoV HKU1, OC43, NL63, and 229E were stabilized as described previously [13], codon-optimized for mammalian expression, and His-tagged for purification. The constructs for the antigens were ordered from GeneArt, and the plasmids were made and transfected in mammalian cells using Expifectamine (ThermoFisher, A14525) according to the manufacturer's protocol.

### Enzyme-linked immunosorbent assay (ELISA)

The immunoglobulin (Ig)G assays were performed as described previously [12]. For the IgA, Maxisorp NUNC-immuno flat-bottomed 96-well plates (Thermo Scientific) were coated with 2 µg/ml of spike antigens of the four endemic coronaviruses and SARS-CoV-2 at 37°C for 1 hour, then washed three times in 0.1% Tween 20 (Sigma) and once in phosphate-buffered saline (Sigma), placed in wash buffer, and blocked with Blocker™ Casein (Thermo Fisher) for 1 hour. Human NP/OP in viral transport media were heat-inactivated for 1 hour at 56°C, and samples were diluted 1: 1 in Blocker™ Casein, added to both receptor binding domain and spike-coated plates, and incubated for 2 hours at room temperature. After washing four times with wash buffer, a 1: 1000 dilution of horseradish peroxidase-conjugated goat antihuman IgA antibody (Sigma) in wash buffer was added to plates, incubated for 1 hour at room temperature, washed, and added with o-phenylenediamine dihydrochloride substrate (Sigma) for color development for 10 minutes. Plates were read on an Infinite® 200 PRO microplate reader (TECAN) at 492 nm, and optical density (OD) values for each sample acquired for analysis. For SARS-CoV-2, IgG seropositivity was defined as a sample OD greater than two times the negative OD. For the HCoV assays, negative OD was defined as three times the blank OD. The cut-offs were defined following a validation exercise during the development of the ELISA, with 174 SARS-CoV-2 PCR-positive Kenyan adults and a panel of sera from the UK National Institute of Biological Standards and Control (NIBSC) and 910 serum samples from Kilifi drawn in 2018, prepandemic [12]. In the World Health Organization-sponsored multilaboratory study of SARS-CoV-2 antibody assays, our results were consistent with the majority of the test laboratories [14].

### Pseudo-neutralization assay

We adapted a lentivirus-based SARS-CoV-2 pseudovirus assay, developed by the Craig laboratory, with minor modifications [15]. Under biosafety level 2 laboratory (BSL2) conditions, the three plasmids, coding the murine leukemia virus (MLV), MLV-gag/pol backbone, luciferase, and full-length spike protein were co-transfected into HEK293T cells using polyethylenimine (PEI) (Polysciences, 24765-1) to produce single round of infection competent pseudoviruses. The medium was changed 24  hours after transfection, and the supernatant containing MLV-pseudotyped viral particles was collected 72  hours after transfection, aliquoted, and frozen at -80°C for the neutralization assay. Virus infectivity was determined by titration on HeLa angiotensin-converting enzyme (ACE2) stable cells as described before [16], and the dilution of pseudoviruses giving >20,000 relative light units (RLU) was selected for assaying. To test for neutralization of the cross-reactive antibodies, we selected the 30 highest responders and 15 of the lowest responders of SARS-CoV-2 spike protein in both serum and NP/OP ELISA. All serum and NP/OP samples were heat-inactivated at 56°C for 1 hour. In sterile 96-well plates (Corning, 353077), 50 μl of the virus was immediately mixed with 50 μl of serially diluted (2 ×) serum or NP/OP, starting at 1: 50 and 1: 1 dilution, respectively, and incubated for 1 hour at 37°C to allow antibody neutralization of the pseudotyped virus. In all, 10,000 HeLa-ACE2 cells/well (in 100 μl of media containing 20 μg/ml dextran) were directly added to the antibody-virus mixture. Plates were incubated at 37°C for 72 hours. After the infection, HeLa-ACE2 cells were lysed using lysis buffer (25 mM glycylglycine pH 7.8, 15 mM MgSO4, 4 mM EGTA, 1% Triton X-100, Promega, E2661). Luciferase intensity was then read on a luminometer with luciferase substrate according to the manufacturer's instructions (Promega, E2650). The percentage of neutralization was calculated using the following equation: 100  ×   (1 – [RLU of sample – average RLU of background/average of RLU of probe alone – average RLU of background]), where background was the cell only control and probe was the virus and cells without serum or NP/OP. As a positive assay control for seroneutralization, a pool of convalescent serum from 50 individuals with confirmed COVID-19 was included. As part of validating the pseudovirus assay, 21 SARS-CoV-2 PCR-positive Kenyan adults and a panel of sera from the UK NIBSC, and 30 serum samples from Kilifi drawn in 2018 and nonreactive to SARS-CoV-2 spike were analyzed with expected results (Supplementary Figure S1).

### Statistical analysis

Data analysis was conducted using R v4.1.0. ELISA responses were compared using Student's *t*-test and Wilcoxon signed rank test. Data were considered statistically significant at *P <0.05, **P <0.01, ***P <0.001, ****P  0.0001, and not significant. To estimate the inhibitory dilution 50(ID_50_), the dilution curves were fit to each sample and the mean of each group, with the neutralization percentage modeled using a five-parameter log-logistic function of the dilution factor based on the Reed-Muench method [17]. This yielded an ID_50_ value for each sample and group, where the curves were fit using drc package v3.0-1 in R v4.1.0 [18]. Samples that did not show a dilution response because of no neutralization were not assigned an ID_50_ value.

## Results

**Figure 1:**
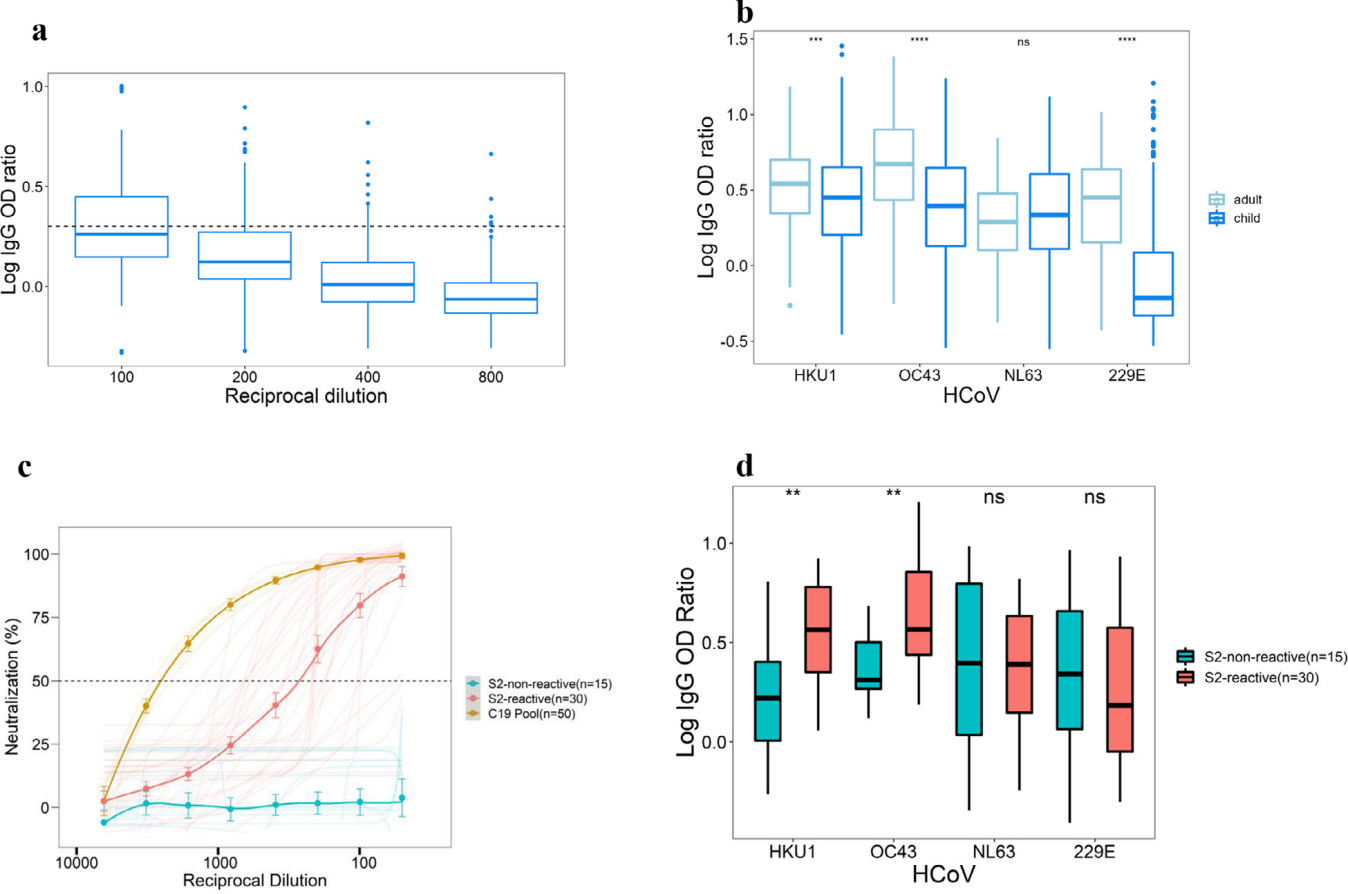
Reactivity of prepandemic and COVID-19 serum to coronaviruses spike and to SARS-CoV-2 pseudo-type. (a) Enzyme-linked immunosorbent assay to SARS-CoV-2 spike antigen with prepandemic human serum (n = 220) showing high cross-reactivity which decreases with increasing fold dilutions. Dotted line shows cut-off for positivity. (b) Enzyme-linked immunosorbent assay to HCoV spike antigens with the prepandemic human serum showing responses among adults (n = 195) and children (n = 431). There were significantly higher responses in adults than children with all HCoV spike antigens except HCoV-NL63 spike. (c) Pseudotyped SARS-CoV-2 neutralization using the selected SARS-CoV-2 spike (S2) reactive IgG (n = 30) and nonreactive IgG (n = 15) samples are shown. There was neutralization with the S2-reactive samples, mean ID_50_ of 1:251 compared with mean ID_50_ of 1: 2461 of COVID-19 pooled (C19 pool) convalescent serum used as assay-positive control but no neutralization with S2-nonreactive IgG samples. (d) There were significantly higher IgG responses for HCoV-HKU1 and OC43 for S2-reactive than S2-nonreactive sera but no significant difference for NL63 and 229E. HCoV, human coronaviruses; Ig, immunoglobulin; ns, not significant; OD,optical density.

**Figure 2:**
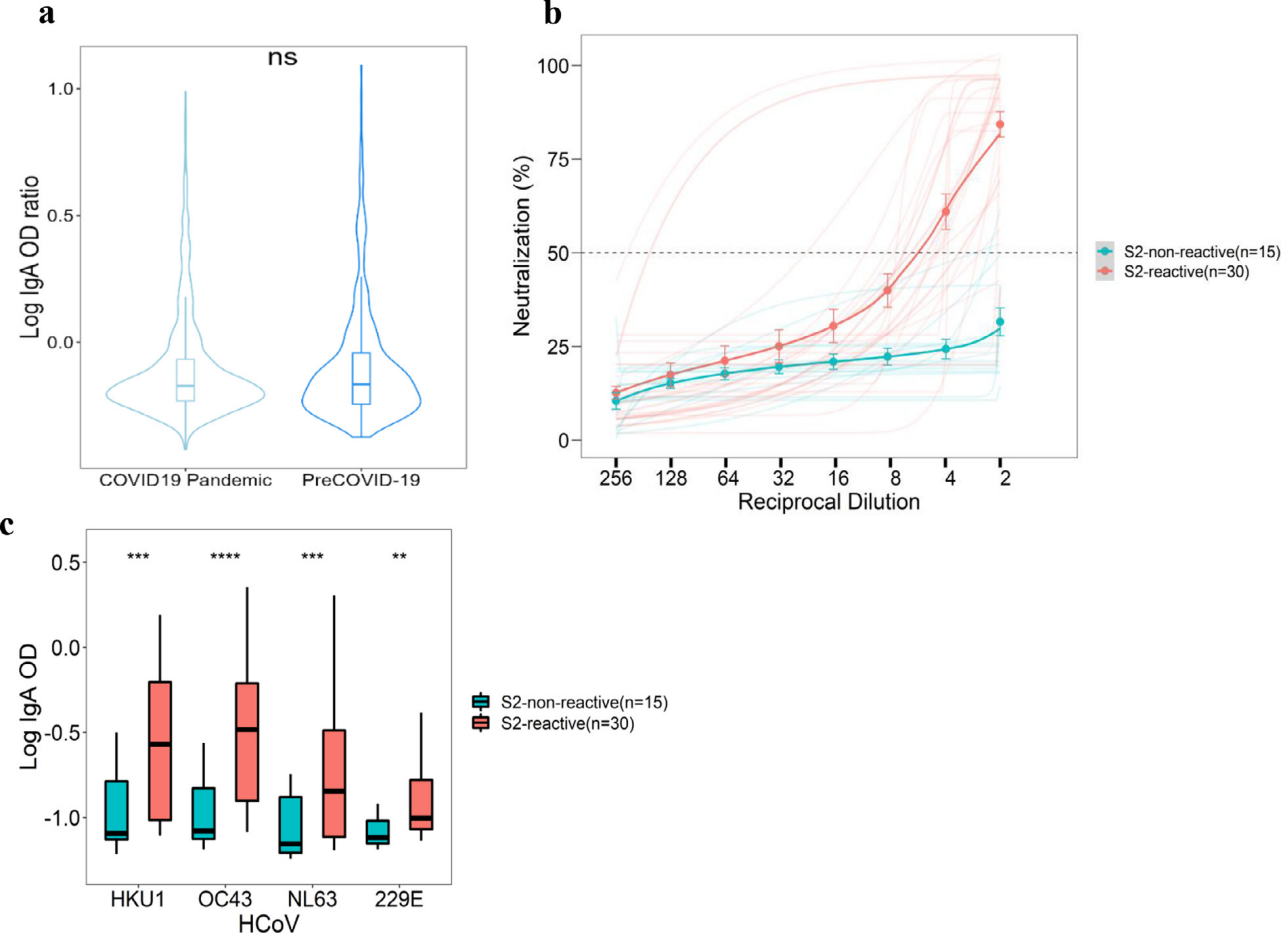
Reactivity of prepandemic and COVID-19 naso-oropharyngeal swabs to coronaviruses spike and to SARS-CoV-2 pseudo-type. (a) Enzyme-linked immunosorbent assay to SARS-CoV-2 spike antigen with prepandemic and pandemic nasopharyngeal swabs with SARS-CoV-2-positive reverse transcriptase-polymerase chain reaction result showing high cross-reactivity with no significant difference between the two sample sets. (b) Pseudotyped SARS-CoV-2 neutralization using the selected SARS-CoV-2 spike (S2) reactive IgA (n = 30) and nonreactive IgA (n = 15) samples are shown. There was neutralization with the S2-reactive samples, mean ID50 of 1:5.9 but no neutralization with nonreactive IgA (n = 15) samples. (c) There were also significantly higher responses of S2-reactive IgA samples than the S2-nonreactive IgA samples among the four endemic HCoV. HCoV, human coronaviruses; Ig, immunoglobulin; ns, not significant; OD, optical density.

SARS-CoV-2 spike reactive IgG antibodies were found in 93/220 (42.3%) Kenyan prepandemic serum samples at 1: 100 of dilution, but these levels reduced with increasing dilutions, and at 1: 800 dilution, only 5/220 (2.5%) were above our positivity cut-off (Figure 1a). Furthermore, there were pre-existing IgA antibodies reactive to SARS-CoV-2 spike in prepandemic NP/OP samples at similar levels to those in NP/OP samples collected from patients with positive PCR results for SARS-CoV-2 on diagnostic testing (Figure 2a).

We hypothesized that the apparent prepandemic immunity was driven by the presence of HCoV antibodies in these samples, and that the responses would be stronger toward the closely related betacoronaviruses (HKU1 and OC43) than the alphacoronaviruses (NL63 and 229E). To investigate this, we designed constructs for full-length trimeric spike proteins for HCoV HKU1, OC43, 229E, and NL63 as described previously [13] and expressed the proteins in mammalian cells and confirmed expression of the specific recombinant proteins on SDS-PAGE and Western blot (Supplementary Figure S2a). We developed ELISAs using the recombinant antigens and validated the responses using convalescent serum from seven individuals with specific RT-PCR-confirmed HCoV infections (Supplementary Figure S2b). IgG antibodies to the infecting HCoV were detected with strong OD responses by ELISA (log of the area under the curve >10); although, there were additional responses at lower levels (log of area under the curve <10) directed at other HCoV not detected by PCR (Supplementary Figure S2b). Next, we investigated the presence of the HCoV in 626 prepandemic serum samples and found IgG antibodies to all the four HCoV, and three of the four HCoV had significantly higher responses in adults than in children (*P* <0.001; Figure 1b).

To investigate the neutralization functions of the pre-existing cross-reactive IgG and IgA antibodies, we determined the median response and designated samples above the median as “SARS-CoV-2-S-reactive” and below the median as “SARS-CoV-2-S-nonreactive”. We then randomly selected 30 SARS-CoV-2-S-reactive IgG samples and 30 SARS-CoV-2-S-reactive IgA samples. As controls, we randomly selected 15 SARS-CoV-2-S-nonreactive IgG samples and 15 SARS-CoV-2-S-nonreactive IgA samples and performed a neutralization assay using the wild-type pseudo-SARS-CoV-2. Of the 30 SARS-CoV-2-S-reactive IgG samples, 29 exhibited neutralizing activity against pseudo-SARS-CoV-2, with a mean ID_50_ of 1: 251, whereas all the 15 SARS-CoV-2-S-nonreactive IgG samples showed no neutralizing activity (Figure 1c). Compared with a pool of 50 convalescent serum collected from individuals with confirmed COVID-19 (ID_50_ of 1: 2461), their neutralizing titers were about 10-fold less (Figure 1c). Khoury *et al.* [19] have estimated the 50% protective neutralization titer of most of the SARS-CoV-2 convalescent serum to be between 1: 10 and 1: 1200 in *in vitro* neutralization experiments, suggesting that the titers observed in our study could be protective. To further establish whether these antibodies could be protective, we determined the levels of SARS-CoV-2 binding IgG antibodies by normalizing both the reactive and nonreactive SARS-CoV-2 IgG binding antibodies using the World Health Organization standard, NIBSC 20/136. We found that 5/30 (16.7%) of the SARS-CoV-2-S-reactive IgG samples had greater than the 60-154 binding antibody units/ml suggested to be protective for IgG binding antibodies [20], whereas 23/30 (76.7%) had levels considered SARS-CoV-2-seropositive according to the positivity (>32 binding antibody units/ml) threshold suggested by Chibwana *et al.* [21] (Supplementary Figure S3). One of the 15 SARS-CoV-2-S-nonreactive IgG samples was seropositive, but none reached the protective threshold. Interestingly, when we compared the ELISA responses between the 30 SARS-CoV-2-S-reactive IgG and 15 SARS-CoV-2-S-nonreactive IgG samples, only HKU1 (*P* <0.001) and OC43 (*P* <0.01) had significantly different responses, implying that the neutralization of SARS-CoV-2 was mainly associated with these two HCoVs in serum, which are both betacoronaviruses as SARS-CoV-2 (Figure 1d).

In contrast, there were significant differences between 30 SARS-CoV-2-S-reactive IgA samples and 15 SARS-CoV-2-S-nonreactive IgA samples among the four HCoVs, implying a nonbetacoronaviruses-specific neutralization effect (Figure 2c). However, 28/30 (93.3%) of SARS-CoV-2-S-reactive IgA samples exhibited neutralizing activity to the reference pseudo-SARS-CoV-2 virus, with mean ID_50_ of 1: 5.9, whereas the SARS-CoV-2-S-nonreactive IgA samples did not neutralize (Figure 2b).

## Discussion

COVID-19 morbidity and mortality has been surprisingly low in sub-Saharan Africa (SSA) compared with the rest of the world, despite the burden of infectious diseases, malnutrition, and insufficient health care [22]. The low burden has been variously hypothesized to be due to Africa's favorable weather; timely mitigation measures; younger population structure; high exposure to infectious diseases, such as malaria, resulting in immune priming and production of protective cross-reactive T cells and antibodies from bacteria and endemic HCoVs, such as HKU1, OC43, NL63, and 229E [22,23]. We have previously reported SARS-CoV-2 spike reactive antibodies in prepandemic serum in a section of our in-house ELISA validation panel [12]; here, we report the presence of SARS-CoV-2 neutralizing serum IgG (mean ID_50_ of 1: 251) and mucosal IgA (mean ID_50_ of 1: 5.9) antibodies reactive to HCoV spike proteins in prepandemic samples. Consistent with our IgG data, Ng *et al.* [7] reported HCoV-induced IgG antibodies capable of neutralizing SARS-CoV-2 in the prepandemic samples from the UK, with neutralizing titers ranging from 1: 100 to 1: 3000 dilution. In contrast to our study, Ng *et al.* [7] observed higher cross-reactive antibodies in children (21/48 [44%]) than in adults (16/302 [5.3%]) than the children (19/95 [20%]) and adults (74/125 [59.2%]) in our study. Some studies have reported similar findings as our study and attributed the results to continued boosting after reinfection and provided an explanation to better protection in children as not the high levels of mature class-switched IgG and IgA antibodies but higher levels of immature HCoV IgM, which are more adaptable in antigen recognition and fragment crystallizable (Fc) responses [24,25]. However, there are several studies with contrasting data on HCoV antibody levels in adults versus children; age could therefore be a confounder [24,25]. Nevertheless, neutralization by prepandemic sera was attributed to antibodies against antigenic epitopes conserved within the spike S2 subunit of SARS-CoV-2 and HCoV, especially HKU1 and OC43 [7]. SARS-CoV-2 neutralizing antibodies (ID_50_ ranging from 1: 10 to 1: 100) in the prepandemic sera, targeting both S1 and receptor binding domain have also been reported in children and adults in the United Kingdom and illustrates these as additional targets for cross-neutralization [26]. These studies suggest protective role of pre-existing HCoV immunity to the clinical course of COVID-19 after SARS-CoV-2 infection, and this might be the case in our population; although, the 42.3% prevalence of cross-reactive antibodies does not fully account for the 92.4% asymptomatic individuals observed in our population [27], implying that other factors contribute [22]. Notably, Tso *et al.* [28] have reported a higher prevalence of HCoV in SSA than in the United States and associated the lower mortality and morbidity observed in SSA with prepandemic HCoV serological cross-reactivity. Apart from humoral immunity, SARS-CoV-2-specific T cells from prepandemic individuals have been reported to cross-react with sequences from endemic coronaviruses, plasmodium, and commensal bacteria, implying that the latter may also contribute to the protective properties of the prepandemic samples [23,29]. In contrast, a recent study involving hospitalized patients with COVID-19 associated pre-existing HCoV antibodies with severe and fatal outcomes of COVID-19 and attributed the effect to the original antigenic sin phenomenon [11]. However, the study included only hospitalized patients sampled at a single time point, making it impossible to determine the level of the previous HCoV immunity [11]. In fact, a 7-month longitudinal cohort involving asymptomatic and participants with mild/moderate symptoms showed that a previous HCoV exposure had a protective effect against SARS-CoV-2 infection and disease [30]. Nevertheless, knowing the duration of HCoV protective immunity to SARS-CoV-2 infection and COVID-19 will be the key to the understanding of the role of HCoV on COVID-19 epidemiology and pathology at the population level.

Prepandemic breast milk IgA antibodies binding to both SARS-CoV-2 and HCoV spike proteins have been reported in mothers in Uganda and the United States [31], but to the best of our knowledge, our study is the first report of neutralizing mucosal IgA antibodies to SARS-CoV-2 in prepandemic NP/OP samples. However, neutralizing mucosal IgA antibodies after SARS-CoV-2 infection have been reported elsewhere, with better neutralizing capacities than monomeric IgA and IgG in the circulation and providing heterologous protection [32,33]. Apart from acting at the primary SARS-CoV-2 invasion sites, mucosal IgA exists in a dimeric form, which has a better antigen binding capacities and can perform both nonspecific (immune exclusion) and specific neutralization and Fc-mediated immune functions [33,34].

We have reported results of mucosal IgA and serum IgG in prepandemic samples from two distinct populations. It would have been better to compare the two antibody classes in the corresponding samples. Therefore, in the absence of corresponding samples in these retrospective samples, our study is limited in drawing inferences from the two populations about the likely behavior of the two classes of antibodies. Furthermore, failure to measure antibodies to SARS and Middle East respiratory syndrome (MERS), presents another limitation because the antibodies are also cross-reactive to SARS-CoV-2 and may contribute a proportion of the responses we have observed. However, SARS and MERS are rare in our setting and therefore widespread responses are unexpected [35,36]. Nevertheless, teasing out the virus-specific responses from a mixture of antibodies would require adsorption of the antibodies with purified spike antigens from the specific coronaviruses, which we have not performed in the current study due to the limited quantities of the retrospective samples.

Overall, our data provide evidence of functional cross-reactive antibodies in prepandemic samples from an African population and suggests an additional explanation for why members of this population appear to be less susceptible to severe COVID-19 disease. A full understanding would need a direct comparison to samples from other geographic locations and longitudinal studies measuring HCoV antibodies before SARS-CoV-2 infection and follow-up of the individuals through the pandemic to estimate the percentage of those who were infected with SARS-CoV-2, percentage of those who were sick and admitted to hospital, and percentage of those who died.

## Declaration of Competing Interest

The authors have no competing interests to declare.

## Appendix Supplementary materials

Image, application 1
