# Supplementary material for: Serum immunoglobulin G and mucosal immunoglobulin A antibodies from prepandemic samples collected in Kilifi, Kenya, neutralize SARS-CoV-2 in vitro
Source: Int J Infect Dis. 2023 Feb;127:11–6. doi: 10.1016/j.ijid.2022.11.041 (PMC9721188; doi:10.1016/j.ijid.2022.11.041)
Supplement: Supplementary file 1 [file mmc1.docx]

**Supplementary figure 1**

**a**


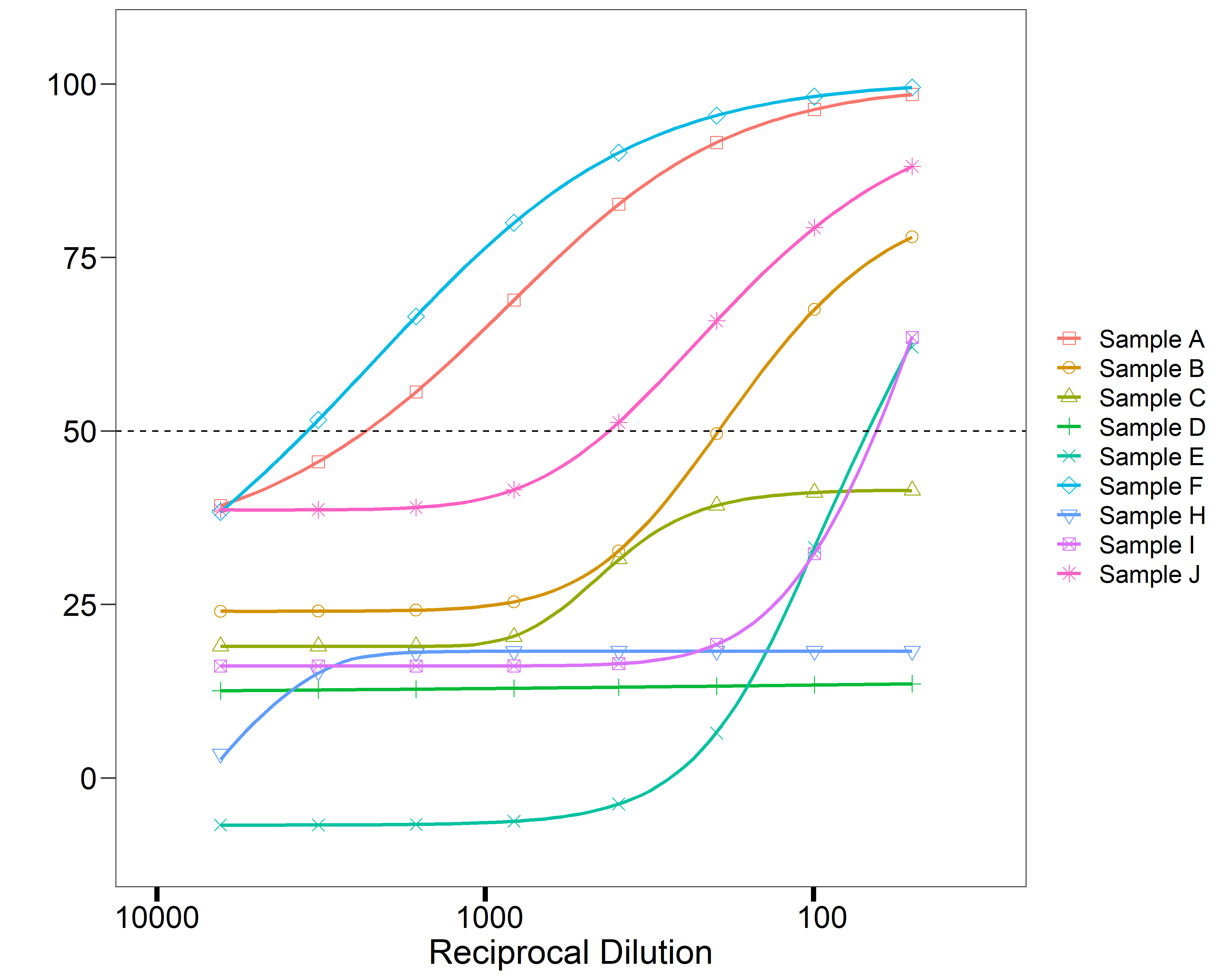


**b**


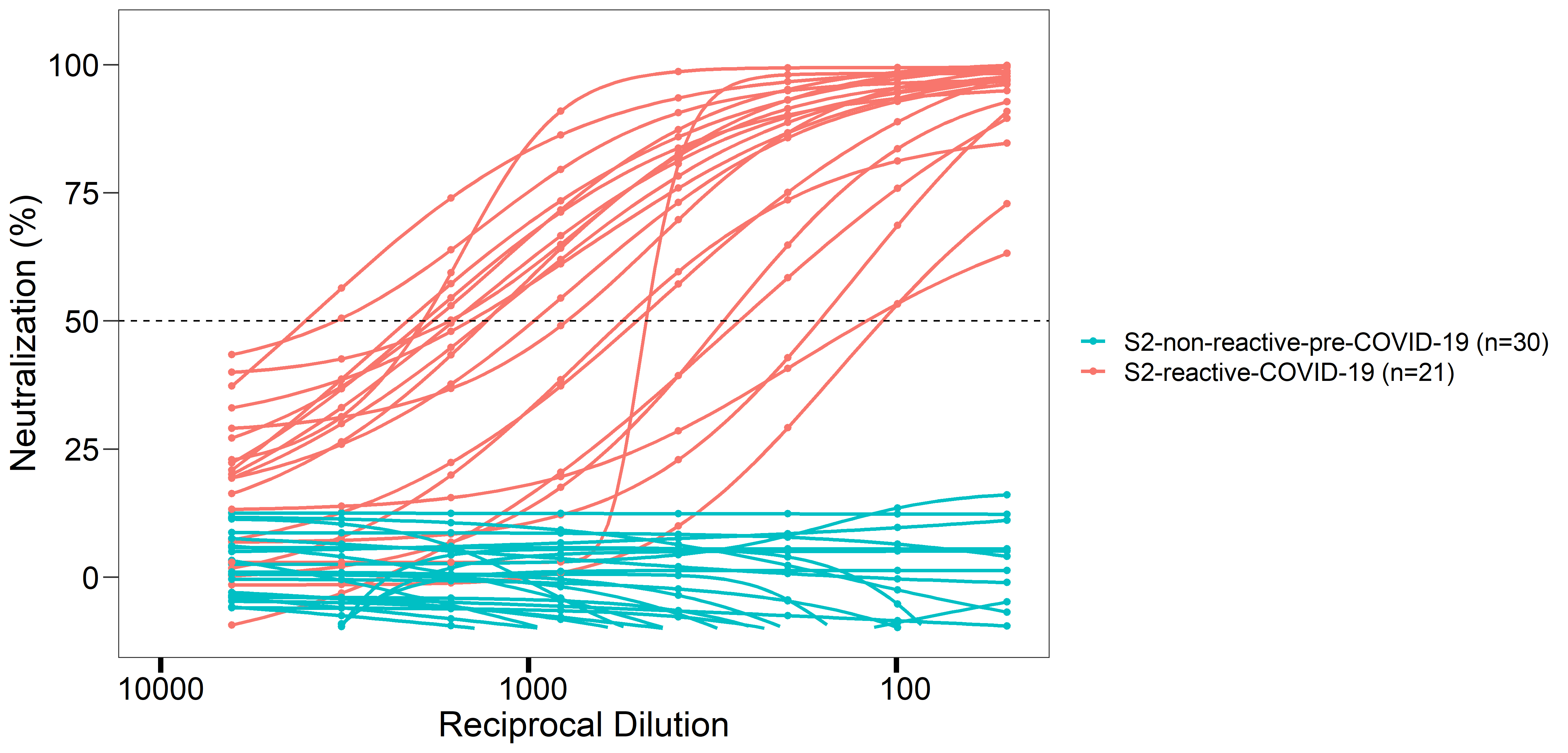


**Fig. S1. Validation of the in-house pseudoviral assay**

1. **SARS-CoV-2 neutralizing titres of the World Health Organization reference panel asembled by the UK National Institute of Biological Standards and Control, as determined in our in house pseudoviral assay.** The expected pseudoviral neutralizing titres from other laboratories were highly concordant with our results. The order of neutralizing titres were as expected, high: sample F>A, mid: J>B, low: E>I, negative: samples C, D and H
2. **SARS-CoV-2 neutralizing titres of selected in-house gold standard poitive and gold standard negative samples.** There were variable SARS-CoV-2 neutralizing titres in the sera collected from 21 individuals with PCR confirmed SARS-CoV-2 and spike ELISA positive (S-2-reactive-COVID-19) but no neutralizing titres in the pre-pandemic sera collected from 30 individuals before the onset of COVID-19 and spike ELISA negative (S2-non-reactive-pre-COVID-19).

**Supplementary figure 2**

**a**


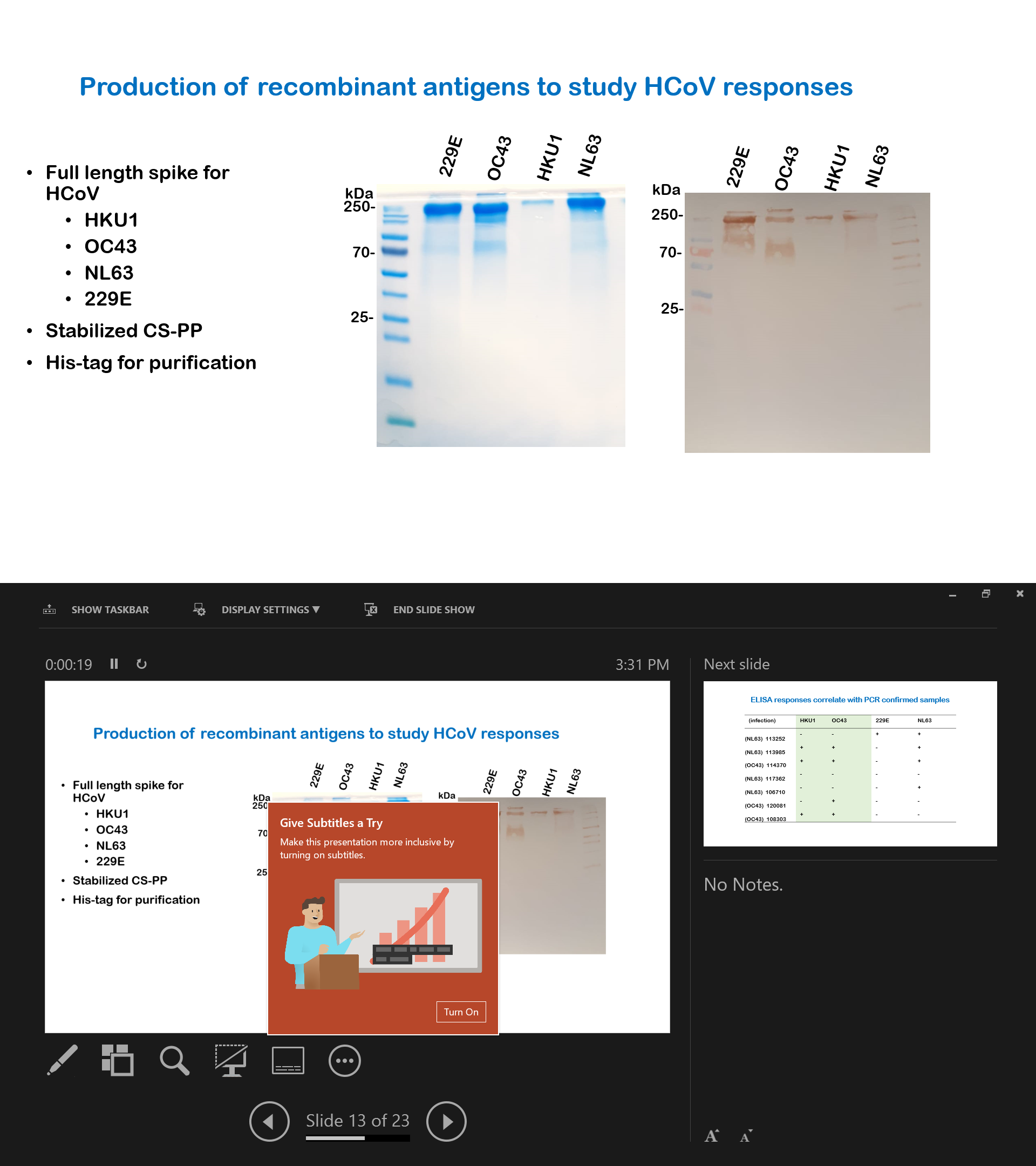


**b**


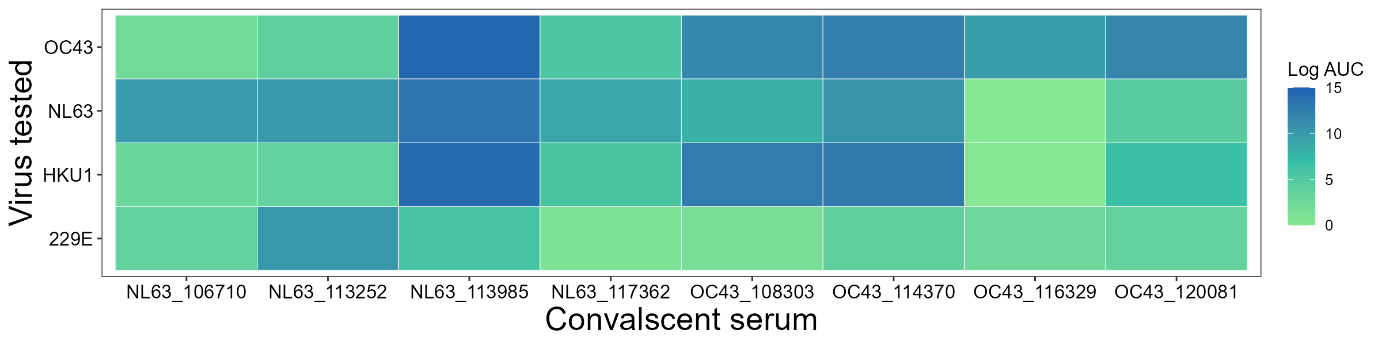


**Fig. S2. Production of recombinant HCoV spike antigens and validation with HCoV convalescent serum**

1. SDS-PAGE and Western blot images of the recombinant HCoV 229E, OC43, HKU1 and NL63 whole spike proteins are shown.
2. ELISA responses to HCoV spike proteins using convalescent serum from individuals with RT-PCR confirmed HCoV infections

**Supplementary figure 3**


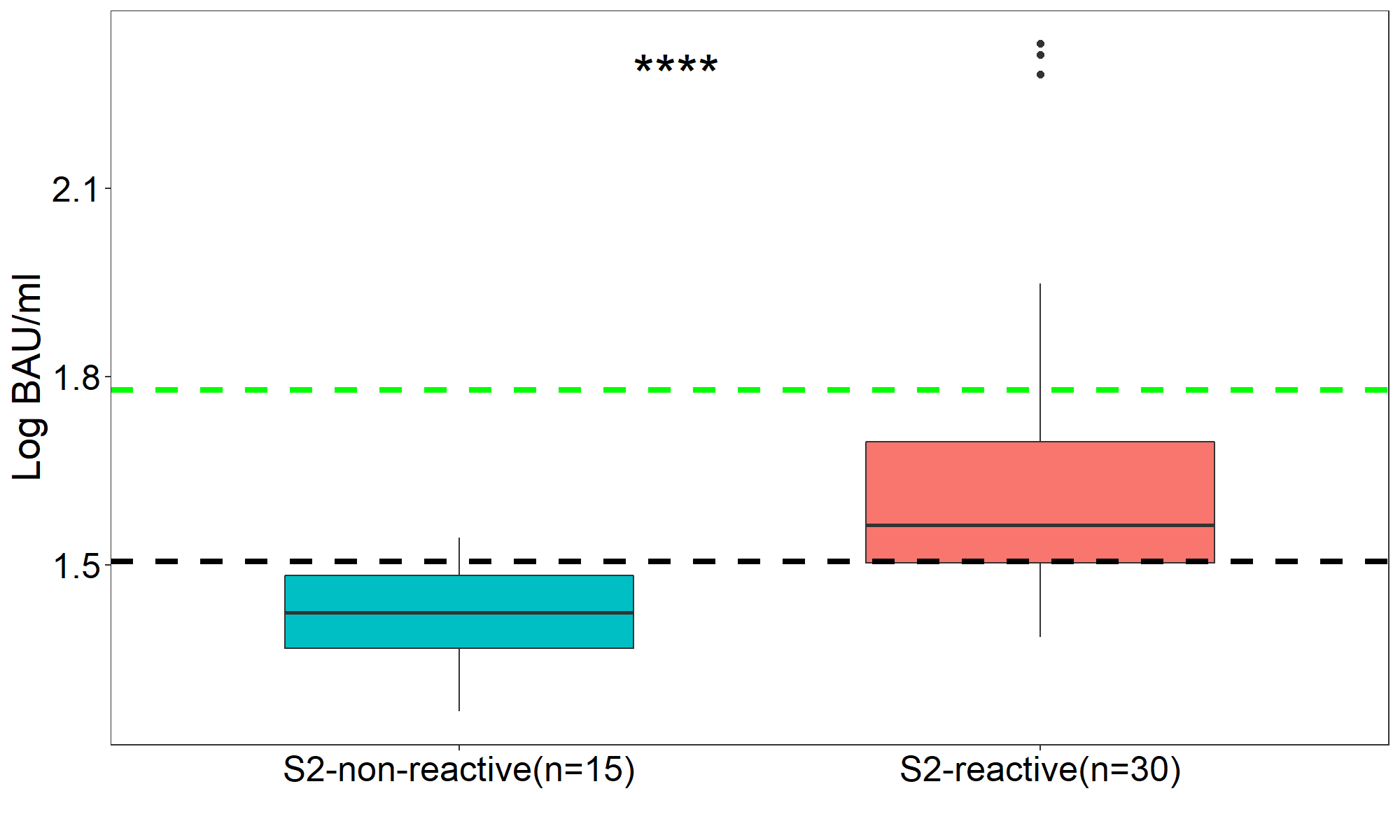


**Fig. S3. SARS-CoV-2 spike binding antibody units for neutralizing and non-neutralizing serum samples**

Binding antibody units to SARS-CoV-2 spike normalized using the WHO standard polyclonal antibody, NIBSC 20/136 reagent for the S2 non-reactive (non-neutralizing) and S2-reactive (neutralizing) samples. Thresholds for SARS-CoV-2 seropositivity (>32 BAU/ml) suggested by Chibwana et al.,2022 and SARS-CoV-2 protective antibodies (60-154 BAU/ml) suggested by Goldblatt et al., 2022 are indicated by black and green doted lines respectively.
